# Supplementary material for: Self-assembly of biomorphic carbon/sulfur microstructures in sulfidic environments
Source: Nat Commun. 2016 Sep 15;7:12812. doi: 10.1038/ncomms12812 (PMC5027620; doi:10.1038/ncomms12812)
Supplement: Supplementary Information — Supplementary Figures 1-4 [file ncomms12812-s1.pdf]

Supplementary Information

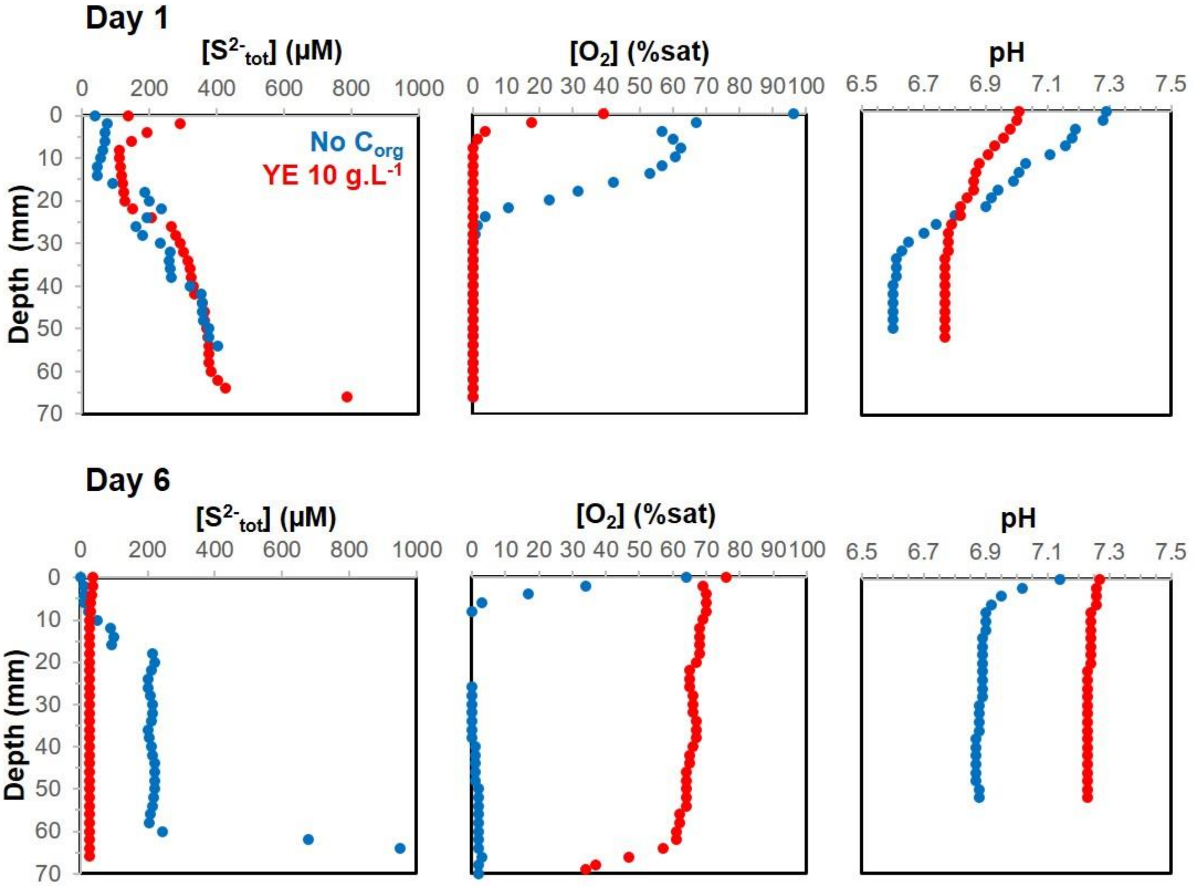

Supplementary Figure 1. Vertical profiles of total sulfide concentration ([S^{2-}_{tot}]), dissolved oxygen saturation ([O_2]) and pH in the over-layer of sulfide gradient tubes. Total sulfide concentrations were calculated using measured H_2S and pH profiles (see Methods section). Profiles were measured after one and six days of experiment in sulfide gradient tubes prepared in the absence of dissolved organics (in blue) or in the presence of yeast extract 10 g.L^{-1} (in red). At the beginning of the experiments (Day 1), sulfide profiles in the presence and in the absence of organics are similar. However, after six days, all sulfide has been consumed in the presence of organics, and oxygen, which is no longer consumed by sulfide oxidation, has diffused back in the tubes. Sulfide is still present in the organic-free gradient tubes.

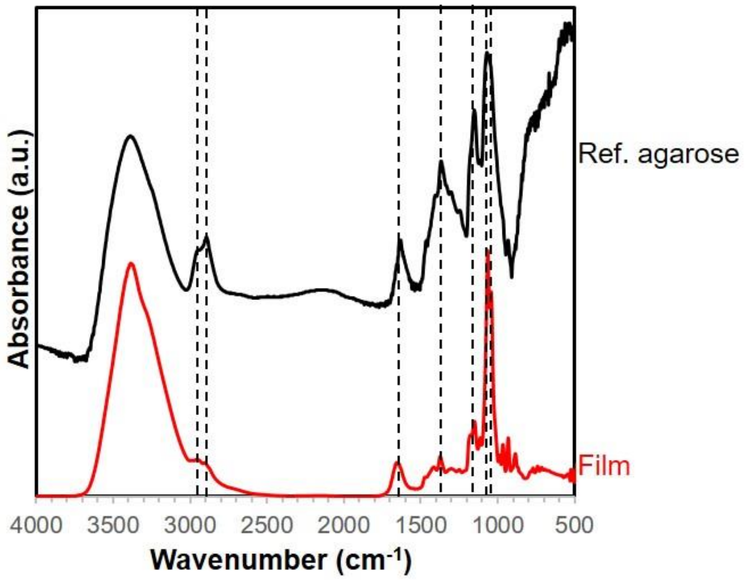

Supplementary Figure 2. Fourier Transform Infrared spectrum of the film formed in the top layer of the sulfide gradient tubes. Comparison with a reference agarose spectrum shows that the film is composed of agarose. The vertical lines correspond to the energies of the main peaks of the agarose spectrum.

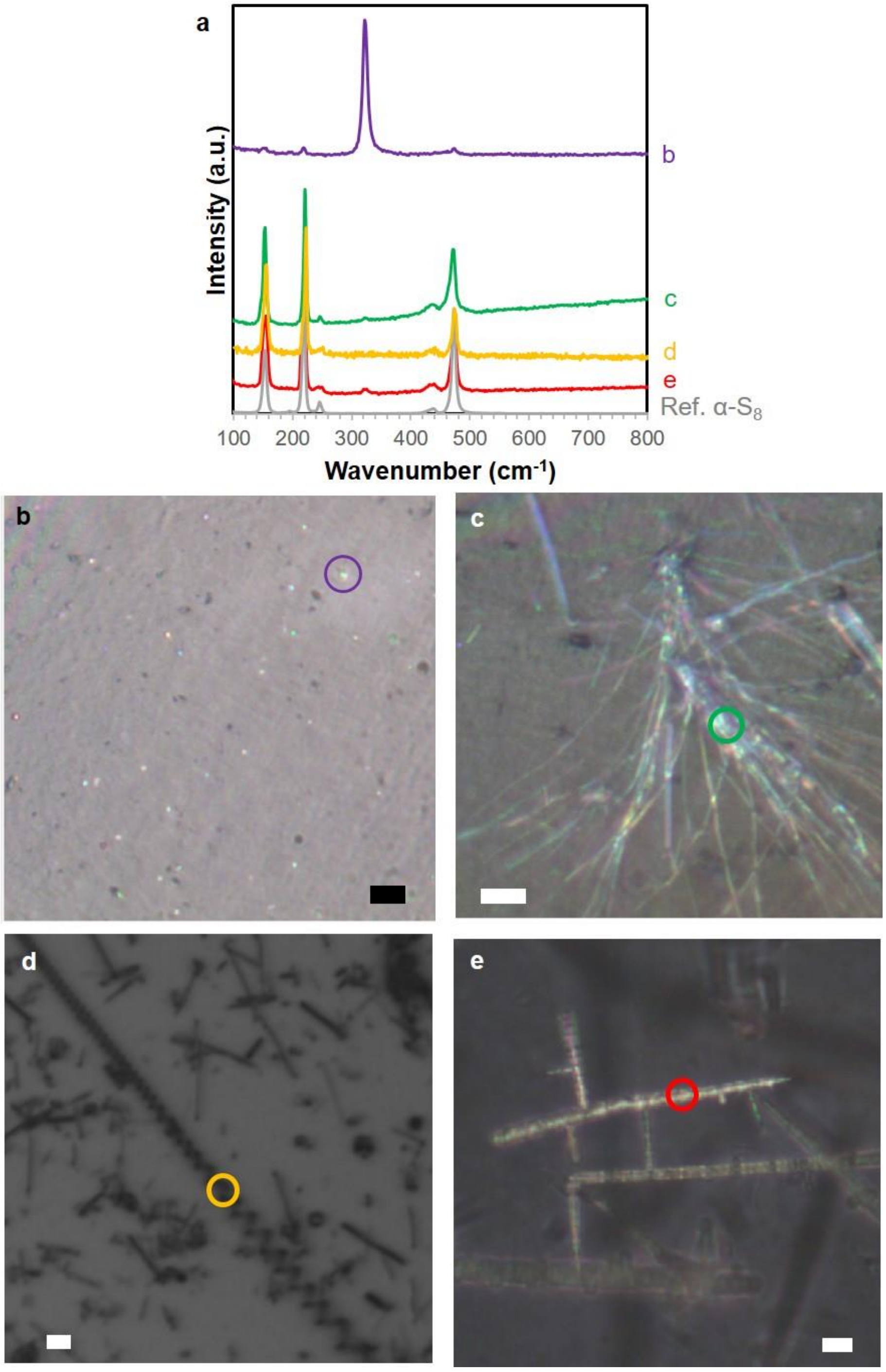

Supplementary Figure 3. Raman spectromicroscopy analyses of C/S microstructures. (**a**) Raman spectra obtained on the areas depicted by the colored circles on images (b-f). The peak at ~322 cm^{-1} corresponds to the CaF_2 substrate on which the samples were deposited for Raman analyses. Images (b-e) correspond to the following conditions: (**b**) No organic carbon; (**c,d**) yeast extract 2 $g.L^{-1}$; (**e**) yeast extract 2 $g.L^{-1}$ and peptone 10 $g.L^{-1}$. The spectrum reference S^0 (cyclooctasulfur α-S_8) is shown for comparison (grey in **(a)**). Scale bars: (**b-e**) 5 µm.

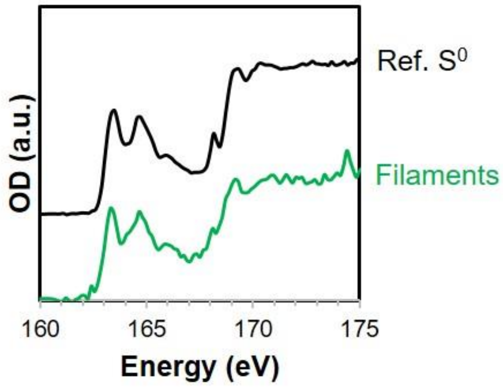

Supplementary Figure 4. S L-edge STXM/XANES spectrum of the filamentous microstructures. The spectrum of a reference elemental sulfur compound (S^0) is shown for comparison.
